# The differential effects of core stabilization exercise regime and conventional physiotherapy regime on postural control parameters during perturbation in patients with movement and control impairment chronic low back pain

**DOI:** 10.1186/1758-2555-2-13

**Published:** 2010-05-31

**Authors:** Ramprasad Muthukrishnan, Shweta D Shenoy, Sandhu S Jaspal, Shankara Nellikunja, Svetlana Fernandes

**Affiliations:** 1 Srinivas College of Physiotherapy and Research Center (SCPTRC), Rajiv Gandhi University of Health Sciences, Mangalore, Karnataka, India; 2 Department of Sports Medicine and Physiotherapy, Guru Nanak Dev University, Amritsar, Punjab, India; 3 Kasturba Medical College, Mangalore, Karnataka, India

## Abstract

**Background:**

The purpose of the present study was to examine the differential effect of core stability exercise training and conventional physiotherapy regime on altered postural control parameters in patients with chronic low back pain (CLBP). As heterogeneity in CLBP population moderates the effect of intervention on outcomes, in this study, interventions approaches were used based on sub-groups of CLBP.

**Methods:**

This was an allocation concealed, blinded, sequential and pragmatic control trial. Three groups of participants were investigated during postural perturbations: 1) CLBP patients with movement impairment (n = 15, MI group) randomized to conventional physiotherapy regime 2) fifteen CLBP patients with control impairment randomized to core stability group (CI group) and 3) fifteen healthy controls (HC).

**Results:**

The MI group did not show any significant changes in postural control parameters after the intervention period however they improved significantly in disability scores and fear avoidance belief questionnaire work score (P < 0.05). The CI group showed significant improvements in Fx, Fz, and My variables (p < 0.013, p < 0.006, and p < 0.002 respectively with larger effect sizes: Hedges's g > 0.8) after 8 weeks of core stability exercises for the adjusted p values. Postural control parameters of HC group were analyzed independently with pre and post postural control parameters of CI and MI group. This revealed the significant improvements in postural control parameters in CI group compared to MI group indicating the specific adaptation to the core stability exercises in CI group. Though the disability scores were reduced significantly in CI and MI groups (p < 0.001), the post intervention scores between groups were not found significant (p < 0.288). Twenty percentage absolute risk reduction in flare-up rates during intervention was found in CI group (95% CI: 0.69-0.98).

**Conclusions:**

In this study core stability exercise group demonstrated significant improvements after intervention in ground reaction forces (Fz, Mz; g > 0.8) indicating changes in load transfer patterns during perturbation similar to HC group.

**Trial registration:**

UTRN095032158-06012009423714

## Background

A plausible contributing factor to CLBP is poor control of trunk muscles to the exigencies of day-to-day activities. Core stabilization exercises are focused to address inter-segmental stability by facilitating neuromuscular control in the lumbar spine.

Studies have reported that specific stabilization exercises reduces pain and disability in chronic but not in acute low back pain and can be helpful in the treatment of acute low back pain by reducing recurrence rate [1].

Many EMG studies have reported changes in spinal muscle recruitment patterns after short and long-term specific core stability intervention in patients with CLBP [2,3]. It has been reported that temporal changes in preprogrammed feedforward adjustments[2-4] firing patterns, amplitudes of activation [4] and reorganisation of trunk muscle representation at the motor cortex [5] achieved after specific stabilization exercises focused on transversus abdominis and multifidus co-contraction.

On other hand not emphasizing the local core muscle activation during exercises found with no changes in relative EMG amplitudes of local muscles after 12 weeks of complex stabilization exercise training in CLBP [6].

Studies have advocated efficient neuromuscular control for trunk stability [7], accurate trunk muscle recruitment patterns for controlling spinal load in relevant to given task and posture [8,9] for impaired trunk control[10] and poor balance[11] associated with CLBP. Further correlation between impaired postural control and delayed muscle response time in twelve major trunk muscles was also reported for patients with CLBP [11].

Though stabilization exercises have become a major focus in spinal rehabilitation as well as in prophylactic care such as sports injury prevention [7], the therapeutic evidences in terms of postural control variables were not well documented. Further many randomized controlled trials have comprehensively reported the effects of core stability exercises versus conventional physiotherapy treatment regimes on pain characteristics, recurrence and disability scores in CLBP patients emphasizing patient centred outcomes [12-16]. These studies have addressed the need of homogenous CLBP group for better clinical outcomes. Evaluating postural control parameters such as COP displacements, moments and forces following interventions particularly stability exercises, may provide insight into how this surrogate outcomes are mediated by different subgroups or heterogeneous CLBP patients and identifying subgroups of CLBP patients who are most likely to benefit after particular intervention.

Keeping the view of heterogeneity as a confounding factor in CLBP and to classify the CLBP patients into homogenous groups, O'Sullivan's sub-classification system was adopted i.e., movement and control impairment CLBP groups [15-17]. This heterogeneity in CLBP population could differentially moderate the effect of given intervention on outcomes. Hence in this study treatment interventions were chosen based on sub-grouping of the CLBP patients. Further effort was also made to analyze pattern of pain behaviour engendered by flare-up of symptoms during the intervention period.

The study was done in outpatient departments of affiliated hospitals with little interference to normal physiotherapy practice and with consented participants scheduled for posturography assessments. We aimed to provide results of practical relevance for back rehabilitation in outpatient physiotherapy settings. The derived intention of this study was to determine whether core stability training helps the CLBP patients, the way they respond when they come across unexpected sources in a day-to-day life that displace their body's centre of gravity out of base of support, impinging neutral zone and act as hidden cause of persistent back pain. Hence purpose of the present study was designed to examine the differential effect of core stability exercise training and conventional physiotherapy regime with mobilization on postural control parameters during perturbation induced postural challenge in patients with control impairment and movement impairment chronic low back pain.

## Methods

### Subjects

Patients were recruited from 4 hospitals in Mangalore city, Karnataka, India. CLBP patients, who were referred by general physicians (GP) or self referred, screened by GP over January 2009 to July 2009 for physical therapy outpatient department of SCPTRC, District Wenlock Hospital, ESI Hospital and NMPT Hospital, were screened for eligibility criteria.

Inclusion criteria required subjects to be between the ages of 18 to 55 years and to have primary compliant of back pain, nonspecific in nature, who had a minimum of one previous episode of LBP necessitating alteration in normal activities or for which medical care/intervention has been sought - less than 1 year, patients in the sub-acute or chronic stage (onset of their current episode of pain not less than 8 weeks) and pain rating on visual analog scale less than 6.

Exclusion criteria were: Red flags (Warrants physician referral and constant supervision): evidence of cauda equina compression, non mechanical LBP and clinical presentations suggestive of acute objective motor radiculopathy or nerve root compression, Surgical: abdominal surgery within the past 12 months, any spinal surgery, limb surgery. Medical: systemic illness, neurological or muscular degenerative disorders Others: pregnancy or less than 1 year postpartum, any orthopaedic impairments, fractures, peripheral vascular disease, subjects with body mass index more than 27, subjects with central nervous system impairments and any respiratory or cardiovascular impairment affecting perturbation trial.

### Therapists

Twelve musculoskeletal specialized physical therapists (Mean age(SD): 34.45(1.5) with mean(SD) clinical experience of 7.9(1.3) years participated in the rehabilitation of CLBP subjects. Two sports physiotherapists with eight and half years (SD:1.4) of mean clinical experience (One physiotherapist trained under Curtin university and another physiotherapist worked in back rehabilitation setting along with first physiotherapist for 4 years) did CLBP sub-classification based on guidelines provided by O'Sullivan [15,16] and they were not provided treatment tasks of CLBP in the trial. This classification system was based on set of substantially reliable [18] essential characteristics proposed by Dankaerts and O'Sullivan et al [18] i.e., "pain with minimal radiation and absence of impaired movement of the symptomatic segment in the painful direction of movement or loading (based on clinical joint motion palpation examination)". If hypomobility or the presence of impaired movement was judged at involved segment, the subject was categorised into MI group [18,19].

The study was planed to examine the conventional physiotherapy with back ergonomic advice commonly used in Indian physiotherapy outpatient settings compared to core stability exercises combined with ergonomic advice. Detailed core stability and conventional therapy stage-wise regimes were made as a protocol in catchment hospitals to treat the participants before starting the trial i.e., 6 months. Participants also participated for a larger study investigating motor control dysfunction during varieties of perturbation. The study was approved by ethical committee of SCPTRC.

**Figure 1:**
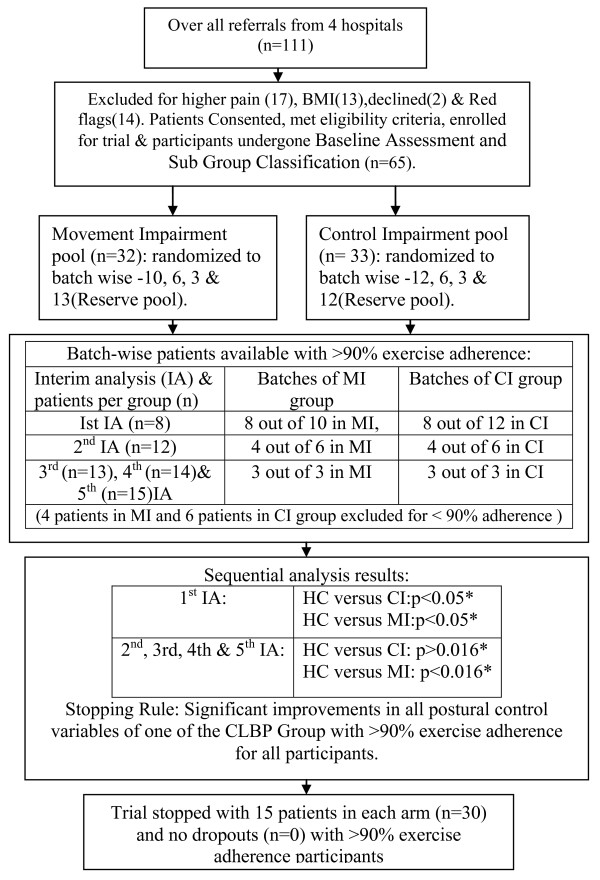
**Flowchart of participant recruitment, retention & interim analyses**; *p < 0.05 and p < 0.016: Armitage-McPherson adjusted critical p values based on no. of interim analyses (k) i.e., for first and fifth interim analyses respectively.

Random number table was used to generate randomization sequence. Pre-numbered identical envelopes were used for allocation concealment. The trial process for all patients was depicted in figure 1.

Keeping the view of extreme poor adherence to 4 days per week exercise regime and larger clinical load in Indian physiotherapy settings, the trial was planned to sequentially and interimly analyse the effect of eight weeks intervention with equal size of participants in each group. It was planed to terminate the trial once significant improvements obtained from any of CLBP group after intervention in surrogate forceplate parameters. Stopping rule of the trial was with 90% of adherence for exercise regimes of each participant in each group and significant improvement in any of CLBP group compared to healthy controls.

Interim statistical analysis was performed for initial group of 8 patients in each arm with 90% exercise adherence out of 10 in MI group and 12 in CI group. Further batches consisted of 6 and 3 patients were also generated sequentially using random numbers and interim analyses were done as depicted in flow-chart. All consented, eligible participants during the trial period were kept for subsequent inclusion for the trial as proceeds as reserve pool.

A batch of four patients fully adhered with 90% exercise regime were included for 2^nd ^interim analysis and further batch of 3 patients completed the 90% of exercise regime respectively added for 3^rd^, 4^th ^and 5^th ^interim statistical analyses. In 2^nd ^interim analyses 2 patients were excluded for poor exercise adherence in MI and CI group respectively.

### Outcome measures

To obtain reliable and valid scores of disability, fear avoidance belief and pain characteristics, we used Ronald Morris Disability Questionnaires (RMDQ), [20-22], Fear Avoidance Belief Questionnaires (FABQ), [23,24] and Chronic Pain Grade Questionnaires(CPQ), [25-27] respectively. Participants were asked to rate level of pain on 100 mm horizontal line i.e., visual analog scales (VAS) anchored by the statement "no pain" on the left and "the most intense pain imaginable" on the right [28]. VAS pain scale has demonstrated good reliability [29] and concurrent validity [30] when compared with other methods of pain measurement.

Questionnaires were given to participants and asked fill and return with in two days before and after intervention. Necessary items in the questionnaires required help were assisted by junior physiotherapists those had no clinical responsibilities in carrying out the trial.

The CPQ was used to detect resolution, improvement, persistence and aggravation and the recurrence component was modified to assess flare-up of an episode during intervention by adopting minimal important change of 2 units on a 0-10 units pain intensity scale. During the intervention period all patients asked to inform any flare-up of pain by using questions one, two and three of CPQ and used as flare-up recurrence in this study. Two units rise from the baseline during intervention was considered as flare-up recurrence. This was done to find out the variation of pain patterns during the intervention period.

Bertec force (60 cm × 40 cm) plate was used with sampling Frequency of 1000 Hz with auto zero facility. The time set was made into 5 seconds and filter was setup made it to 500 Hz. In Acquired channel set of ACQ (Data Acquisition Software Digital Acquire), forces [(Fx, Fy, Fz (Newton)], Moments [(Mx, My Mz (Nm)] and COPx, COPy (meter) parameters were chosen.

Subjects stood barefoot on the force platform comfortably and their feet 25 cm apart and medial malleolus of both feet was at same level (toe out 10^0^). They were instructed to stand still and their visual gauze was fixed at 5 m in front of them on wall. Perturbation was delivered by a mechanical pendulum from behind [31]. The three and half kg was standardized as the minimum amount of weight required to bring perturbation or keeping 5% body weight for each subjects. When in its vertical position, the pendulum was aligned with the position of the subject's heel. The pendulum moved 20 degree from the vertical line at a distance of 90 cm away from the subjects at mid thoracic level to produce necessary perturbation.

Trials were made to standardize the weight to bring adequate perturbation on trunk after the impact. The weights were necessarily increased based on the participant body weight and to produce quantifiable postural adjustments. Subjects were instructed to use preferred leg to move on space to adjust the perturbation to stabilize while maintaining stance leg on the forceplate. They were advised to bring the leg back to original stance as early as possible and not to step on. Large stumbling reactions were repeated and no subjects reported pain during perturbation trial.

The perturbation, which produced the change in force, i.e., sudden shift or increase in forces of Fy axis (AP direction in our study) was monitored to find the exact perturbation time or impact time. The perturbation was identified by defined millisecond of sudden force shift from Y-axis by considering AP direction of perturbation with minimal 2SD from base line on force plate data for a 50 ms period.

From perturbation time to 2000 ms recording was used to calculate COP displacements. Anterior and posterior translations of COPy and medial and lateral translations of COPx were negated to produce net difference in COPy and COPx displacements respectively. Peak values of forces (Fx, Fy, Fz) and peak values of moments (Mx, My, Mz) were obtained from 5000 ms of storage data form the forceplate after plotting into MATLAB. Three trial mean values were accumulated each above parameters. Two forceplate sessions were performed before and after intervention period of 8 weeks.

### Core stabilization regime

A basic outline of the anatomy of the various local and global muscles and the differences in their function was given before the start of the program. During the 8 week intervention period, 4 day per week with initial fourteen guided training sessions took place emphasizing core muscle co-contraction, each lasting 45 min.

The 8 week treatment protocol was divided into 3 phases. In the first phase of the training, attention was focused on facilitating isolated local muscle activity with emphasis on continuation of normal breathing. Subsequently, the hold time and the number of repetitions were increased, and subjects were trained to maintain these contractions in various postures (four-point kneeling, supine, prone, sitting and standing).

As commonly prescribed in Indian settings, traction and interferential therapy were also given based on clinical judgment of treating therapist (27%). Once an accurate and sustained contraction of the local muscles was achieved in different postures (10 to 15% MVC, 10 contractions with 10-s holds), the exercises progressed to the second phase which involved applying low load to the muscles through controlled movements of the upper and lower extremities. The main aim during the third phase was to integrate these low grade static contractions along with the normal static and dynamic functional tasks so that these contractions become involuntary. 92% exercise adherence was recorded in this group. At the end of 8^th ^week 53.3% of CI group was at third phase, 33.3% at second phase and 13.3% remained at first phase.

### Conventional regime

The subjects in the CI group were given conventional physiotherapy and basic strengthening exercises in accordance with their clinical symptoms.

Based on physical examination, in phase I, MI group subjects received mobilization or manipulation for 3 minutes on the lumbar vertebral segment under therapist's judgment (72% MI group) and during the course of intervention on demand basis lasting approximately 3-5 minutes (7%). Traction and interferential therapy were also combinatively used based on clinical judgment of treating therapist (32%). Rate of perceived exertion scale was used to monitor level of exertions (PRE) during strengthening exercises, ranged from 6 to 9, 10 to 15 and 16-20 for respective phases.

43% of patients underwent hyperextension exercise program as a main mode of treatment. 6% patients underwent flexion exercise program as main mode of treatment. They also trained such as isometric trunk lifts and pelvic tilts. In phase II, all patients were trained with classic abdominal, back extensor endurance training and relaxation training initiating range of motion exercises without provoking pain. Phase III was progressed into tougher exercises such as abdominal crunches and trunk lifts added with gym exercises without provoking pain. 91% exercise adherence was recorded in this group. At the end of 8^th ^week 66.66% of MI group was at third phase, 26.66% at second phase and 6.66% at first phase.

The treatment team was blinded to the study only until treatment. Progression of patients in both groups was decided by the treating physiotherapist. Both groups of patients were taught back ergonomics care lessons, model demonstration to use safer lifting techniques in back care classes during first week of intervention. Commonly all exercise sessions approximately lasted up to 45 minutes. The treatment guidelines adapted from the studies of O'Sullivan [15,19], Kuukkanen and Malkia et al [32] for CI and MI group respectively.

During first week of intervention program, more priority was given to back education classes and pain reduction in both groups. At 8^th ^week, all patients instructed to perform exercise at home and to report any pain flare-ups if any and given scheduled appointment for forceplate testing after 8^th ^week.

### Statistical analysis

We calculated relative risks, the number needed to treat, absolute risk reduction, and their 95% confidence intervals using CPQ for the proportion of patients based on resolution, improvement, persistence, aggravation, and with flare-up recurrence during the intervention period. For effect size, we calculated Hedges'g and 95% confidence interval using mean difference of pre and post intervention scores.

Independent t-test was used to analyse the difference between CLBP group and healthy controls. The Armitage-McPherson adjustment was used to correct nominal critical P value (0.05) into adjusted critical p value (0.016) [33]. This adjustment was done because of the repeated interim analyses in our trial [33,34].

Since five interim analysis-significance tests were performed to monitor and attain the significant improvements in postural control variables (p < 0.05), the p value was adjusted to 0.016 and same set of data were used for the first interim analysis of scores of disability, fear avoidance belief and pain characteristics (Adjusted p < 0.05). Paired t-test was used between pre and post intervention postural control parameters of CLBP groups with P < 0.016 significance.

For intention to treat analysis we assumed any participant not available at any point of assessment scores to have not improved. Statistical analysis was performed using the SPSS version 11.0. Statistical analysis was performed by another statistician who was not involved on trial for sample size determination and randomization.

## Results

The groups were similar in age, sex distribution, height, weight, RMDQ and FABQ on baseline assessments. Follow up was 100% for the primary clinical outcomes and surrogate postural control outcomes. Fifteen (9 M, 6 F) CLBP patients with [Mean (SD)] age (yrs) 34.21(8), height (cm) 171.54(8.8), weight (kg) 72.01(11.5) and VAS(mm) 3.2(1.2) participated in MI group. Fifteen (8 M, 7 F) CLBP patients with [Mean (SD)] age (yrs) 36.21(6), height (cm) 173.54(6.8), weight (kg) 75.01(9.5) and VAS(mm) 3.9(0.8) participated in CI group. They were classified into extension pattern (n = 8), flexion pattern (n = 4), lateral shift pattern (n = 2) and multidirectional pattern (n = 1) based on set of essential characteristics proposed by Dankaerts and O'Sullivan et al[19] for O'Sullivan's motor control impairment classification system [15,18]. Healthy age matched control were recruited with [Mean (SD)] age (yrs) 36.21(9.2), height (cm) 174.54(5.8), and weight (kg) 78.01(6.5).

### Comparison of CPQ values between CI and MI groups

#### Flare-up

The risk of flare-up recurrence in CI group was 0.06 compared to 0.26 in MI group. 20% absolute risk reduction of flare-up during intervention period was noted in CI group. A Relative Risk of 0.25 indicates flare-up event is less likely to occur in the CI group than in the MI group. In CI group 6.6% patients developed flare-ups compared to 26.66% in MI group and the number need to treat (NNT) for one extra patient to reduce the flareup back pain was 5 (CI: 2.2 to 17.6).

#### Aggravation

CI group demonstrated 6% absolute risk reduction in aggravation of back pain compared to MI group (CI: -5.96% to 19.29%).

#### Persistence

For calculating persistence of back pain one patient who developed aggravation was not excluded from the MI group keeping the view of not to inflate the negative outcome in MI group.

In MI group 33.33% patients persisted with back pain where as 6.67% of CI group persisted with back pain after the intervention period. The absolute risk reduction for persistence of back pain, is 26.67 percent in CI group (CI: -0.32% to 53.66%) and NNT was 4 (CI: 1.9 to 309.3). This indicates that one in every 4 patients will benefit from the core stability exercises after intervention.

#### Improvement

In MI group 46.67% of population improved while compared to 40% of CI group subjects those received the core stability exercises. The difference, the absolute risk increase, was 6.67 percent in CI group (-28.72% to 42.05%) and the NNH was 16 and this indicates one in 16 will be harmed by core stability exercise in this study (CI: 2.4 to 3.5).

#### Resolution

In MI group 86.67% of CLBP patients were not shown any resolution compared to 46.67% of CI group. The absolute risk reduction was 40% (95% CI: 9.45% to 70.55%). The NNT was 3 and this indicates that one in every 3 patients will benefit from the core stability exercises (95% CI: 1.4 to 10.6).

### Comparison of RMDQ scores between CI and MI groups

Pre and post intervention RMDQ scores between CI and MI group, was compared using Paired't' test. Standardized effect sizes (Hedges's g) were then estimated in those cases where group differences were significant (p < 0.05) or wherever necessary.

Though the disability scores were reduced significantly in CI group (P < 0.05), g = 1.15, 95% of CI: 0.38 to 1.93) and MI groups after intervention (p < 0.05, g = 0.73, 95% of CI: -0.01 to 1.47), however, the mean difference scores between CI and MI groups after intervention were not found significant (p < 0.51, g = 0.24, 95% of CI: -0.48 to 0.95).

### Comparison of FABQ scores between CI and MI groups

Similar trend was observed in FABQ scores also in CI and MI group patients in work and physical activity scores. The MI group [Work score: g = 0.66, 95% CI: -0.07 to 1.4. Physical activity score: g = 0.82 95% CI: 0.08 to 1.57] had medium to larger treatment effect size compared to CI group which had medium effect size [Work score: g = 0.64, 95% CI:-0.09 to 1.37 Physical activity score: g = 0.65, 95% CI: -0.08 to 1.39] at 0.05 level significance.

Post intervention scores of work and physical activity of FABQ between groups found non-significant (g = 0.1, 95% of CI: -0.58 to 0.85 and 0.2, 95% of CI: -0.51 to 0.92 for work score and physical activity scores respectively).

### Results of pre and post intervention scores of MI and CI group on postural control parameters

Hedges'g was calculated for confidence interval 95% of mean difference of pre and post groups. Paired't' test was used for statistical significance. The CI group showed significant improvements in Fx (p < 0.013, g = 0.88, 95% of CI: 0.13 to 1.62), Fz (p < 0.006, g = 1.02, 95% of CI: 0.26 to 1.78), and My (p < 0.002, g = 1.18, 95% of CI: 0.40 to 1.95) after 8 weeks of core stability exercises.

The MI group did not show any significant changes in postural control parameters after the intervention period. Nonetheless an attempt was made to examine the effect size of non significant MI group parameters. Surprisingly, Fx (p > 0.016, g = 0.25, 95% of CI: -0.47 to 0.97), Fz (p > 0.016, g = 0.32, 95% of CI: -0.40 to 1.04) and Mz (p > 0.016, g = 0.28, 95% of CI: -0.44 to 1) parameters had shown smaller improvements in postural control parameters.

### Results of post intervention scores of MI, CI and HC groups on postural control parameters

HC group postural control parameters were analyzed independently with pre and post postural control parameters of CI and MI group. This revealed the significant improvements in postural control parameters in CI group compared to MI group indicating the specific adaptation to the core stability exercises in CI group.

Preliminary analysis with pre intervention scores of MI with HC group revealed non significant changes in Fx, Mz, COPx and COPy variables (p < 0.016) and while comparing HC versus CI group, Mx, COPx and COPy postural control parameters were found non-significant (p < 0.016). Post intervention values of back pain groups compared with HC group revealed significant changes persisted even after 8 weeks of intervention in MI group in Fz, Mx and COPy parameters. However CI group demonstrated similar values close to healthy controls with non significant changes(p > 0.016) in all postural control parameters indicating substantial evidence of specific postural adaptation towards core stability training with good exercise adherence.

## Discussion

The main goal of this research was to determine whether core stability therapy would improve postural control variables. The results support our hypothesis that core stability substantially improves postural control parameters in people with chronic low back pain particularly in CI CLBP group.

There were greater improvements in the Mx, COPx and COPy variables of postural control components during perturbation in the CI group that received core stability therapy than in MI group those received conventional therapy compared to HC group. However MI group persistently demonstrated abnormal postural responses during perturbation and thus could not improve the postural control parameters compared to HC group (Fz, Mx and COPy). This finding clearly indicates specificity of spinal stability exercises on improvement of postural control variables in CI CLBP population.

The interesting finding observed in our study was that improvements observed on all postural control parameters of CI group similar to HC group. The CI group demonstrated significant improvements in Fx, Fz, My (p < 0.016) variables and MI group demonstrated no significant improvements in between pre and post intervention scores even at unadjusted p value. A substantial improvement in Z axis variables with larger effect sizes during perturbation [(CI: Fz -Mean difference (MD):-32.74, p < 0.006, g = 1, Mz -MD:-0.573, p < 0.034, g = 0.8), (MI: Fz -MD:-12.78, p < 0.298, g = 0.32, Mz -MD:-0.366, p < 0.438, g = 0.28)] was observed between pre and post intervention scores of CI group. This phenomenon indicates the efficient changes related to load transfer and weight distribution patterns of CI group patients. The changes in y-axis, (the direction of perturbation) i.e., anterior-posterior axis, and the significant improvement found in this axis indicates the CI group's increased ability to respond anticipated perturbation in our trial after intervention (CI group: obtained p values for Fy, My and COPy: 0.05, 0.002 and 0.06). MI group demonstrated no significant or subtle changes in the direction of perturbation (Fy, My and COPy) indicating the difficulty in encountering postural challenge (p > 0.05).

**Figure 2:**
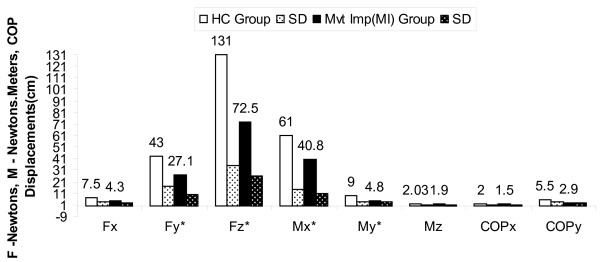
**Forces, Moments & COP displacements during perturbations before intervention in MI group compared to HC Group with independent 't' test results**; * p < 0.016, HC-Healthy controls, MI-Movement impairment CLBP, CI-Control impairment CLBP, SD-Standard deviations.

**Figure 3:**
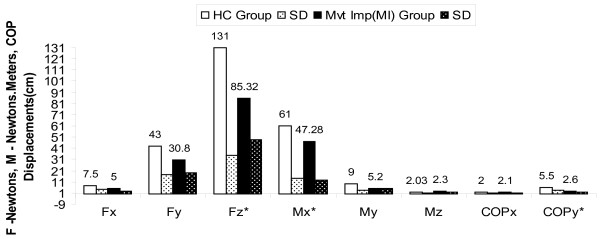
**Forces, Moments & COP displacements during perturbations after intervention in MI group compared to HC Group with independent 't' test results**; * p < 0.016, HC-Healthy controls, MI-Movement impairment CLBP, CI-Control impairment CLBP, SD-Standard deviations.

**Figure 4:**
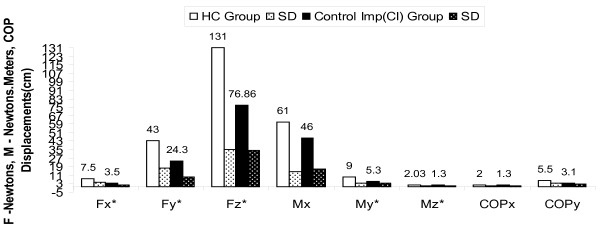
**Forces, Moments & COP displacements during perturbations before intervention in CI group compared to HC Group with independent 't' test results**; * p < 0.016, HC-Healthy controls, MI-Movement impairment CLBP, CI-Control impairment CLBP, SD-Standard deviations.

**Figure 5:**
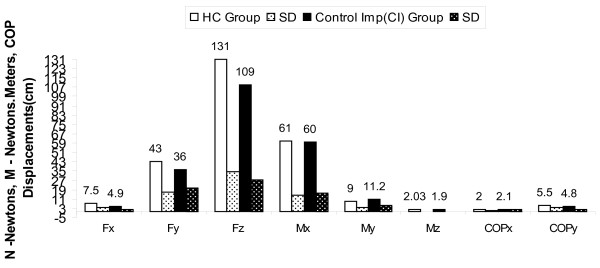
**Forces, Moments & COP displacements during perturbations after intervention in CI group compared to HC Group with independent 't' test results**; * p > 0.016. HC-Healthy controls, MI-Movement impairment CLBP, CI-Control impairment CLBP, SD-Standard deviations.

The net increase in GRF and moment of z axis during perturbation in CI group can be attributed to increased COP excursion velocity during postural adjustments response movements in low back pain patients after stability exercises (Figure 2, 3, 4 and 5). An experimental study reported significant attenuation in GRF in CLBP population walked at their preferred speed but not at fastest speed while comparing with pain free counterparts [35].

The results obtained in our study substantiating the evidence that GRF attenuation during perturbation induced postural voluntary adjustments in CI and MI CLBP patients. Our study further draws the attention on difference in attenuation of GRF in two types of homogenous CLBP population for future studies (p < 0.10) and provides evidence for attenuated GRF can be improved using core stability exercises in CLBP (Figure 2, 3, 4 and 5).

An increase in vertical jump after trunk stability training in athletes reported by Butcher et al. [36] and Milles et al. [37]. They postulated optimized stable lumbo-pelvic base to leg and trunk muscular efforts to load transfer, enhanced neural drive or unknown confounding variable? modulated by stability training; were the causes of increased vertical jump.

Only one aspect of the postural control variable - COPx - did not improve significantly as a result of conventional as well as core stability training or found insensitive for the perturbations given in the study. It appears that medio-lateral postural control adjustment responses may take longer time in back rehabilitation or it may be attributed to wider stance width adopted during our perturbation testing. Henry et al. [38] reported that more trunk displacements in narrow stance due to larger changes in COP oscillations in response to perturbations. They further reported in wide stance, equilibrium control was relied on passive stiffness resulting from changes in limb geometry where as with narrow stance was on active postural strategy regulating loading unloading of the limbs.

It was evident that perturbation testing with wider stance width provided disadvantage to use neuromuscular control strategies in medio-lateral direction and resulted over all decrease in COP displacements in x axis of all participants.

The effect sizes are large and therefore clinically relevant in core stability group. The effect sizes with smaller effect are observed on postural control variables even those who participated in conventional exercise regime. It appears that conventional exercise also had shown cross training effects on postural control parameters. The conventional exercises focused on muscle strength [32] rather than postural strategies may be cause for absence of significant specific postural control improvements in MI group.

Significant improvements were noted in proportion of CLBP population received conventional physiotherapy than core stability regime on CPQ scores. However core stability exercise group demonstrated significant benefits in terms of flare-up of symptoms, aggravation, persistence of back pain and resolution.

The mean difference of disability and FABQ scores between CI and MI groups after intervention were not found significant and indicating similar effects of conventional physiotherapy and core stability on disability and fear of pain for movement during activity.

### Implications

These results indicate that the motor behaviour towards expected perturbations can be changed in CLBP subjects by means of core stabilization training but the duration and long term preservation of these specific adaptations are needed to be studied with special emphasis on postural synergies and its decompositions. These specific adaptations might be small set of muscle synergies that can robustly affect wide range of muscle activation patterns in variable propositions during human postural responses [39].

The preliminary follow up results were promising, a randomly called 5 CLBP participants in CI group retained improved postural control strategies after 3 months of intervention (p > 0.05). This result was supported by an experimental study. Henry and Hodges [2] reported persistence of motor control changes by stability training leads to motor learning of automatic postural control strategies.

### Methodological considerations

To our knowledge, this is the first controlled study examining the differential effects of exercise regimes on postural control variables in CLBP population using combinations of sequential trial design for repeated statistical analysis and randomized patient selection to minimize the threat of internal validity commonly associated with pragmatic trials. This study found evidence indicating that significant improvements in postural control variables specific to CI-CLBP population after core stability regime and not for conventional regime. We statistically minimised the risk of committing false-positive inference (Type I error) that arises from the repeated test of significance by the Armitage-McPherson critical 'p' value adjustment [33,34,40-43]. Caution is warranted in interpreting adjusted p values, as the results from one test are interpreted as being independent of the results form another test [33,34]. Ten consented participants were not included for statistical analysis for poor adherence with exercise regime during interim analysis. Higher number of consented participants and poor exercise adherence was inherent clinical problem and this was cautiously dealt with pragmatic nature of the study design that mimicked routine clinical practice.

However central random allocation to minimize selection bias, adequacy of blinding for the principal outcomes and good reliability of Bertec forceplate with very low crosstalk between forces [44] ensures sufficient internal and external validity to the results.

We intentionally avoided larger group of CLBP population into the trial at a given point of time keeping the view that lesser staffing and larger clinical load. However all consented participants in reserve pool were also given same set of exercises as per their subgroup classification. A cut-off (90%) in exercise adherence was made to get proper data. The lesser exercise adherence had leaded us to plan sequential interim analyses after randomization and that resulted heavy penalty of adjusting critical p value into 0.016 from 0.05 [33,34]. Drop out rates surfaced while analyzing final list of CLBP reserve pool list (drop outs in MI: n = 3 & CI: n = 4) though they were not included for analysis because of stopping rule and the trial found significant improvements in CI group with 15 participants per arm. Nonetheless for detecting 10% deference in the CI group after intervention, a 2-tailed test, an alpha level of .05 and with power of 80%, a total of 22 patients needed per group. This sample size was kept as a futility stopping role of this study, if the emerging data would have suggested that core stability therapy is ineffective in improving postural control variables.

The recurrence section of CPQ was modified to detect flare-up of symptoms. The true recurrence with pain-free status of 1 month can be studied in future studies. The effect of both regimes over movement impairment group can be studied in future as smaller effect sizes were also observed on MI group.

## Conclusions

Though the disability and fear avoidance belief scores were reduced significantly in CI and MI groups, the post intervention scores between groups were not found significant. This indicates that core stability exercises were not superior to conventional physiotherapy exercises in terms of reducing pain and disability. Higher proportions of patients improved in MI group compared to CI group. Core stability exercise group demonstrated and benefited significant improvements in: distribution of ground reaction forces, use of optimized postural adjustments in the direction of perturbation, 20% absolute risk reduction of flare-up during intervention and 40% absolute risk reduction for resolution of back pain after core instability exercises.

## Competing interests

The authors declare that they have no competing interests.

## Authors' contributions

MRP conceived the study, and was responsible for data collection, interim analysis, statistical analysis, and drafting of the manuscript. SSD, NS and SF designed the experimental protocol, collected all data, and helped draft the manuscript. JSS was senior author, providing guidance and advice on all aspects of the study and, in addition, was responsible for the development of the Indian perspective pragmatic design used for data analysis. All authors read and approved the final manuscript and participated in each stages of trial.
